# Supplementary material for: Collateral Circulation and BNP in Predicting Outcome of Acute Ischemic Stroke Patients with Atherosclerotic versus Cardioembolic Cerebral Large-Vessel Occlusion Who Underwent Endovascular Treatment
Source: Brain Sci. 2023 Mar 24;13(4):539. doi: 10.3390/brainsci13040539 (PMC10137090; doi:10.3390/brainsci13040539)
Supplement: Supplementary file 1 [file brainsci-13-00539-s001.zip › supplementary material-Tables.pdf]

Table S1. CE stroke Patient Characteristics at Baseline

Characteristic	All patients (n=77)	Good outcome (n=29)	Poor outcome (n=48)	*P* Value
Age, y; median (IQR)	81.00 (71.50, 86.00)	80.00 (70.00, 80.00)	81.00 (72.00, 85.00)	0.595
Female, n (%)	41 (54.25)	15 (36.59)	26 (63.41)	1.000
NIHSS, median (IQR)	15.00 (10.00, 20.00)	11.00 (7.00, 15.00)	16.00 (13.00, 22.00)	0.003
IV-Tpa, n (%)	22 (28.57)	8 (27.59)	14 (29.17)	1.000
SBP	147.00 (136.00,162.50)	147.00 (135.00,154.00)	150.00 (136.00,166.00)	0.414
DBP	80.00 (71.50, 93.50)	79.00 (70.00, 88.00)	80.00 (75.00, 98.00)	0.739
Risk factors, n (%)				
Smoking	15 (19.48)	8 (27.59)	7 (14.58)	0.235
AF	69 (89.61)	26 (89.66)	43 (89.58)	1.000
Hypertension	62 (80.52)	25 (86.21)	37 (77.08)	0.387
Diabetes mellitus	22 (28.57)	5 (17.24)	17 (35.42)	0.120
Hyperlipidemia	34 (44.16)	12 (41.38)	22 (45.83)	0.814
CHD	39 (50.65)	13 (44.83)	26 (54.17)	0.485
Previous stroke	35 (45.45)	10 (34.48)	25 (52.08)	0.161
Imaging examinations				
IC volume, mL; median (IQR)	39.52 (14.56, 80.77)	27.63 (7.80, 43.54)	54.63 (19.45, 129.24)	0.089
IP volume, mL; median (IQR)	92.73 (53.66, 158.19)	89.67 (18.76,169.20)	97.47 (78.44,140.11)	0.226
MMR, median (IQR)	2.29 (1.41, 4.55)	3.54 (2.23, 7.20)	2.21 (1.07, 4.00)	0.006
FIV, mL; median (IQR)	39.44 (11.05,101.97)	14.00 (3.96,45.15)	113.36 (52.71, 245.90)	<0.001*
ASPECTS, median (IQR)	8.00 (5.00, 9.00)	8.00 (5.00, 8.00)	7.00 (4.00, 9.00)	0.268
4D CTA-CS scores, median (IQR)	2.00 (1.00, 3.00)	3.00 (3.00, 4.00)	2.00 (0.00, 3.00)	<0.001*
CBS, median (IQR)	6.00 (2.50, 9.00)	6.00 (1.00, 9.00)	4.00 (3.00, 9.00)	0.332
Thrombus location, n (%)				0.163
ICA	22 (28.57)	9 (31.03)	13 (27.08)	

Segment M1	33 (42.86)	9 (31.03)	24 (50.00)	
Segment M2	19 (24.68)	10 (34.48)	14 (18.75)	
A1	1 (1.30)	0 (0.00)	1(3.45)	
Tandem occlusion	2 (2.60)	0 (0.00)	2 (4.17)	
Laboratory parameters				
Glucose, mmol/L; median (IQR)	7.20 (6.15, 8.25)	6.50 (5.60, 7.90)	7.60 (6.50, 8.90)	0.199
Creatinine, umol/L; median (IQR)	76.00 (66.00, 88.00)	72.00 (64.00, 80.00)	78.00 (66.00, 91.00)	0.582
Urea, mmol/L; median (IQR)	5.64 (4.28, 7.18)	5.61 (4.52, 7.00)	5.64 (4.08,7.36)	0.099
Uric acid, mmol/L; median (IQR)	324.00 (252.00, 409.25)	317.00 (278.00, 362.00)	345.00 (277.00, 456.00)	0.421
Sodium, mmol/L; median (IQR)	140.00 (138.30, 141.15)	140.10 (136.50, 141.00)	140.00 (138.60, 141.90)	0.097
Potassium, mmol/L; median (IQR)	4.00 (3.70,4.30)	4.10 (3.50,4.30)	4.00 (3.70, 4.20)	0.081
D-dimer; median (IQR)	456.00 (206.50, 914.00)	219.00 (114.00, 446.00)	509.00 (254.00, 1414.00)	<0.001*
Fibrinogen, g/L; median (IQR)	3.01 (2.62, 3.54)	3.05 (2.55, 3.62)	2.94 (2.65, 3.57)	0.232
INR; median (IQR)	0.99 (0.95, 1.10)	1.03 (0.96, 1.10)	0.97 (0.94, 1.05)	0.012
RBC; median (IQR)	4.30 (3.92, 4.78)	4.14 (3.95, 4.50)	4.51 (3.92, 4.86)	0.156
WBC; median (IQR)	7.62 (5.91,9.64)	7.61 (6.07, 9.59)	7.73 (5.80, 9.80)	0.887
BNP, median (IQR)	466.38 (252.33, 738.68)	372.12 (180.66, 467.70)	560.90 (337.00, 867.30)	<0.001*
Time, min; median (IQR)				
Onset to imaging	191.00 (117.50, 294.00)	161.00 (111.00, 271.00)	191.00 (115.00, 288.00)	0.394
Imaging to puncture	73.00 (55.50, 105.00)	70.00 (46.00, 107.00)	74.00 (60.00, 104.00)	0.345

Puncture to recanalization	67.00 (47.50, 100.50)	61.00 (46.00, 90.00)	79.00 (52.00, 120.00)	0.101
Recanalization, n (%)	64 (83.12)	26 (89.66)	38 (79.17)	0.349

CE: cardioembolic; IQR: interquartile range; NIHSS: National Institutes of Health Stroke Scale; SBP: systolic pressure; IV-Tpa: intravenous tissue type plasminogen activator; DBP: diastolic pressure; AF: atrial fibrillation; CHD: coronary heart disease; IC: ischemic core; IP: ischemic penumbra; MMR: mismatch ratio; FIV: final infarct volume; ASPECTS: Alberta Stroke Program Early CT Score; 4D CTA-CS: the modified collateral circulation scoring system on 4D CTA; CBS: clot burden score; ICA: internal carotid artery; M1: M1 segment middle cerebral artery; M2: M2 segment middle cerebral artery; A1: A1 segment anterior cerebral artery; INR: activated partial thromboplastin time; RBC: red blood cell; WBC: white blood cell; BNP: B-type brain natriuretic peptide

Table S2. LAA stroke Patient Characteristics at Baseline

Characteristic	All patients (n=105)	Good outcome (n=70)	Poor outcome (n=35)	*P* Value
Age, y; median (IQR)	76.00 (63.75, 83.25)	65.00 (55.00, 75.00)	81.00 (70.00, 85.00)	<0.001*
Female, n (%)	38 (36.19)	19 (27.14)	19 (54.29)	0.009
NIHSS, median (IQR)	12.50 (7.00, 17.00)	9.00 (6.00, 13.25)	13.00 (9.00, 17.00)	<0.001*
IV-Tpa, n (%)	21 (20.00)	19 (27.14)	2 (5.71)	0.018
SBP	145.00 (132.75,159.25)	142.00 (128.75,150.50)	144.00 (130.00,160.00)	0.352
DBP	80.00 (71.00, 90.00)	80.00 (71.75, 87.25)	80.00 (70.00, 89.00)	0.150
Risk factors, n (%)				
Smoking	38 (19.48)	29 (27.59)	9 (14.58)	0.135
AF	6 (5.71)	0 (0.00)	6 (17.14)	0.001
Hypertension	78 (74.29)	49 (70.00)	29 (82.86)	0.236
Diabetes mellitus	51 (48.57)	31 (44.29)	20 (57.14)	0.300
Hyperlipidemia	49 (46.67)	33 (47.14)	16 (45.71)	1.000
CHD	40 (38.10)	20 (28.57)	20 (57.14)	0.427
Previous stroke	47 (44.76)	29 (41.43)	18 (51.42)	0.406
Imaging examinations				
IC volume, mL; median (IQR)	22.55 (8.99, 57.65)	27.63 (7.80, 43.54)	54.63 (19.45, 129.24)	<0.001*
IP volume, mL; median (IQR)	82.94 (42.92, 128.05)	81.20 (33.40,122.95)	57.37 (35.50, 100.98)	0.168
MMR, median (IQR)	3.04 (1.76, 6.25)	3.54 (2.31, 7.41)	2.25 (1.06, 4.00)	0.017
FIV, mL; median (IQR)	39.44 (11.05,101.97)	16.75 (7.87,41.76)	68.90 (26.03, 195.22)	<0.001*
ASPECTS, median (IQR)	8.00 (6.00, 9.00)	8.00 (6.00, 8.00)	7.00 (6.00, 8.00)	0.643
4D CTA-CS scores, median (IQR)	3.00 (2.00, 4.00)	3.00 (3.00, 4.00)	2.00 (1.00, 3.00)	<0.001*
CBS, median (IQR)	6.00 (3.00, 9.00)	6.50 (4.00, 9.00)	6.00 (1.00, 9.00)	0.641
Thrombus location, n (%)				0.009
ICA	32 (30.48)	24 (34.29)	8 (22.86)	

Segment M1	35 (33.33)	25 (35.71)	10 (28.57)	
Segment M2	20 (19.05)	15 (21.43)	5 (14.29)	
A1	7 (6.67)	4 (5.71)	3 (8.57)	
Tandem occlusion	11 (10.48)	2 (2.86)	9 (25.71)	
Laboratory parameters				
Glucose, mmol/L; median (IQR)	7.50 (6.20, 9.63)	8.05 (6.68, 10.78)	6.70 (5.70, 12.00)	0.017
Creatinine, umol/L; median (IQR)	76.00 (64.75, 88.25)	75.50 (64.00, 88.25)	79.00 (61.00, 105.00)	0.235
Urea, mmol/L; median (IQR)	5.64 (4.23, 7.24)	5.33 (4.23, 6.61)	6.16 (3.86, 9.15)	0.958
Uric acid, mmol/L; median (IQR)	324.00 (252.00, 409.25)	296.00 (240.25, 406.25)	315.00 (247.00, 419.00)	0.288
Sodium, mmol/L; median (IQR)	140.10 (138.18, 142.00)	140.55 (138.58, 143.00)	139.20 (137.80, 141.70)	0.659
Potassium, mmol/L; median (IQR)	4.00 (3.70,4.30)	4.00 (3.78, 4.20)	4.10 (3.80, 4.60)	0.692
D-dimer; median (IQR)	282.50 (140.50, 662.00)	159.50 (74.75, 335.25)	654.00 (245.00, 1309.00)	0.004
Fibrinogen, g/L; median (IQR)	3.05 (2.65, 3.64)	3.06 (2.67, 3.47)	3.41 (2.63, 3.86)	0.966
INR; median (IQR)	0.98 (0.92, 1.05)	0.94 (0.91, 1.01)	1.01 (0.94, 1.05)	0.122
RBC; median (IQR)	4.40 (3.92, 4.85)	4.67 (4.04, 4.95)	4.38 (3.73, 4.63)	0.049
WBC; median (IQR)	8.04 (6.35,10.21)	8.09 (6.39, 9.01)	10.13 (7.04, 11.93)	0.010
BNP, median (IQR)	212.31 (59.87, 477.26)	48.47 (20.72, 130.56)	207.72 (112.93, 338.79)	<0.001*
Time, min; median (IQR)				
Onset to imaging	251.00 (138.25, 449.75)	312.50 (188.75, 595.50)	252.00 (167.00, 511.00)	0.484
Imaging to puncture	76.50 (56.75, 106.00)	84.50 (62.50, 106.75)	74.00 (53.00, 112.00)	0.532

Puncture to recanalization	81.00 (52.00, 135.75)	90.00 (57.00, 138.50)	128.00 (54.00, 175.00)	0.088
Recanalization, n (%)	91 (86.67)	65 (92.86)	26 (74.29)	0.014

LAA: large-artery atherosclerosis; IQR: interquartile range; NIHSS: National Institutes of Health Stroke Scale; SBP: systolic pressure; IV-Tpa: intravenous tissue type plasminogen activator; DBP: diastolic pressure; AF: atrial fibrillation; CHD: coronary heart disease; IC: ischemic core; IP: ischemic penumbra; MMR: mismatch ratio; FIV: final infarct volume; ASPECTS: Alberta Stroke Program Early CT Score; 4D CTA-CS: the modified collateral circulation scoring system on 4D CTA; CBS: clot burden score; ICA: internal carotid artery; M1: M1 segment middle cerebral artery; M2: M2 segment middle cerebral artery; A1: A1 segment anterior cerebral artery; INR: activated partial thromboplastin time; RBC: red blood cell; WBC: white blood cell; BNP: B-type brain natriuretic peptide
