# Supplementary material for: Collateral Circulation and BNP in Predicting Outcome of Acute Ischemic Stroke Patients with Atherosclerotic versus Cardioembolic Cerebral Large-Vessel Occlusion Who Underwent Endovascular Treatment
Source: Brain Sci. 2023 Mar 24;13(4):539. doi: 10.3390/brainsci13040539 (PMC10137090; doi:10.3390/brainsci13040539)
Supplement: Supplementary file 1 [file brainsci-13-00539-s001.zip › supplementary materials-Figures.pdf]

Figure S1. mRS scors at 3 month by 4D CTA in all patients, LAA stroke patients and CE stroke patients

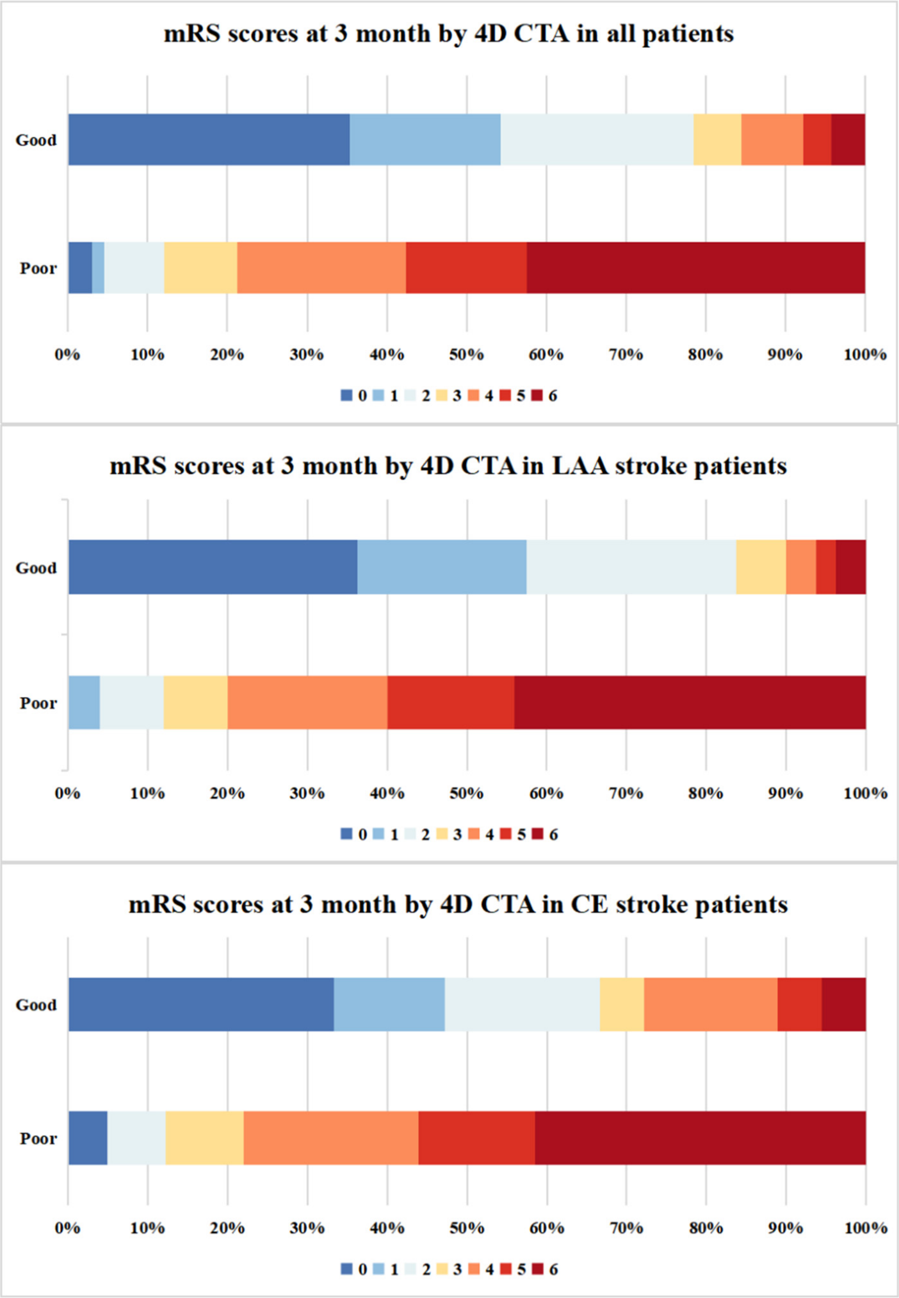

Clinical outcome (modified Rankin scale score at 3 months) in all patients group, LAA stroke group and CE stroke group, stratified by collateral status, including good collaterals group (4D CTA-CS score 3-4) and poor collateral group (4D CTA-CS score 0-2).

Figure S2. mRS scors at 3 month by BNP in all patients and CE stroke patients

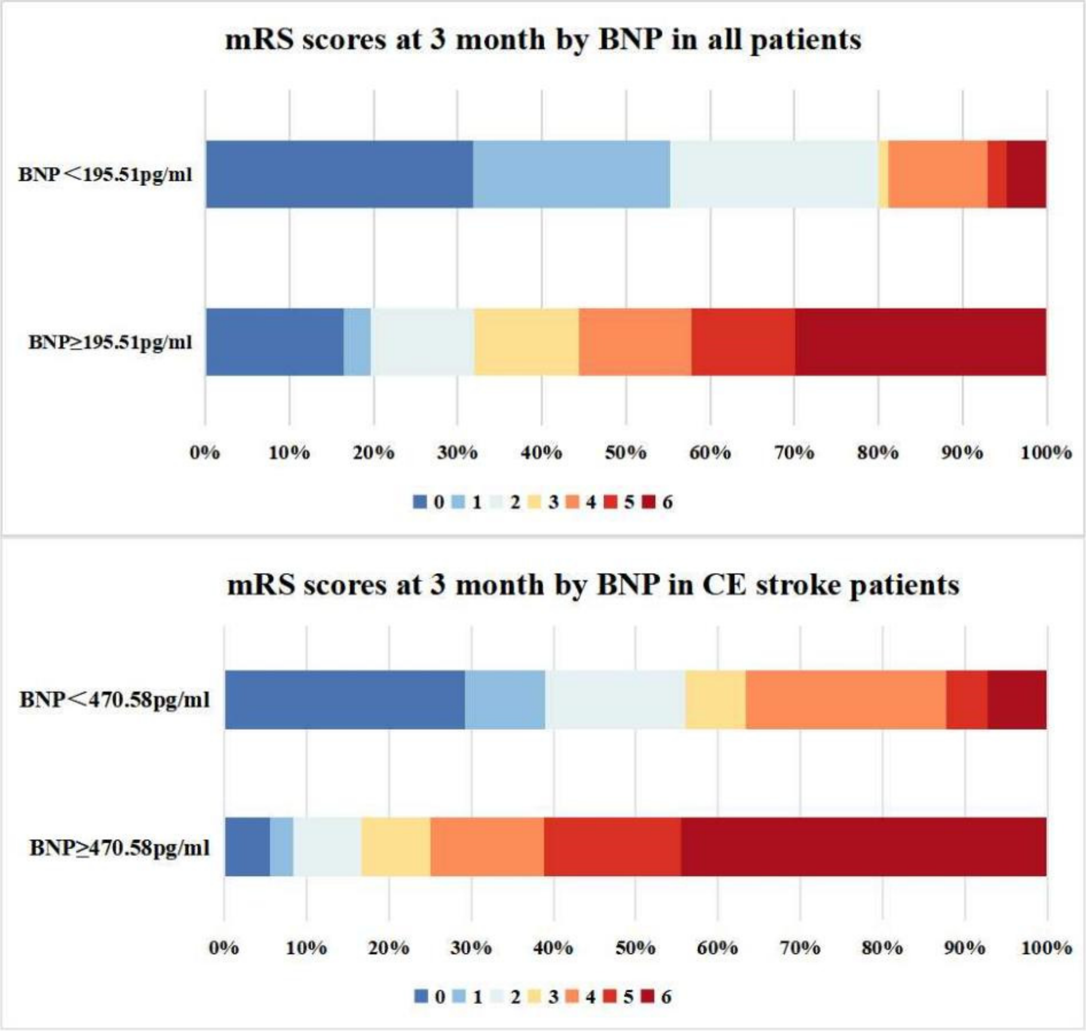

Clinical outcome (modified Rankin scale score at 3 months) in the 2 groups, all patients group and CE stroke group, stratified by the different cut-off value of serum BNP for each group.
